# Do not attempt cardiopulmonary resuscitation decision-making process: scoping review

**DOI:** 10.1136/spcare-2023-004573

**Published:** 2024-03-22

**Authors:** Owen Doody, Hope Davidson, John Lombard

**Affiliations:** 1 Nursing and Midwifery, University of Limerick, Limerick, Ireland; 2 School of Law, University of Limerick, Limerick, Ireland

**Keywords:** Clinical decisions, Ethics

## Abstract

**Objectives:**

To conduct a scoping review to explore the evidence of the process of do not attempt cardiopulmonary resuscitation (DNACPR) decision-making.

**Methods:**

We conducted a systematic search and review of articles from 1 January 2013 to 6 April 2023 within eight databases. Through multi-disciplinary discussions and content analytical techniques, data were mapped onto a conceptual framework to report the data.

**Results:**

Search results (n=66 207) were screened by paired reviewers and 58 papers were included in the review. Data were mapped onto concepts/conceptual framework to identify timing of decision-making, evidence of involvement, evidence of discussion, evidence of decision documented, communication and adherence to decision and recommendations from the literature.

**Conclusion:**

The findings provide insights into the barriers and facilitators to DNACPR decision-making, processes and implementation. Barriers arising in DNACPR decision-making related to timing, patient/family input, poor communication, conflicts and ethical uncertainty. Facilitators included ongoing conversation, time to discuss, documentation, flexibility in recording, good communication and a DNACPR policy. Challenges will persist unless substantial changes are made to support and promote examples of good practice. Overall, the review underlined the complexity of DNACPR decision-making and how it is a process shaped by multiple factors including law and policy, resource investment, healthcare professionals, those close to the patient and of central importance, the patient.

**WHAT WAS ALREADY KNOWN**

- There is significant variability in do not attempt cardiopulmonary resuscitation (DNACPR) decision-making and implementation.
- DNACPR decision-making can be affected by the cultural and ethical backgrounds of decision-makers.
- It is shown that many of the barriers to DNACPR decision-making have been relatively consistent over the past decade.

**WHAT ARE THE NEW FINDINGS**

- Timing and equality in relationships between medical practitioners, patients and those close to patients are essential.
- Decision-making can be affected by legal concerns, economic issues, availability of resources/technologies and perceptions regarding the patient’s quality of life.
- Appropriate and timely communication is vital in discussing diagnosis, prognosis and preferences of each individual patient.

**WHAT IS THEIR SIGNIFICANCE**

- Evidence is needed across a wider variety of healthcare settings, particularly residential care settings.
- There is a need to explore the potential role of technology in health literacy and facilitating informed decision-making on resuscitation status.
- Investment in facilitators of good practice is required (training, professional development) to enhance communication skills.

## Introduction

 Cardiopulmonary resuscitation (CPR) offers the potential to save a person’s life, but in reality, the likelihood of success is generally low for both in-hospital and out-of-hospital cardiac arrest.^1^,^4^ Many people tend to overestimate the effectiveness of the practice and may not be aware of the potential for significant injury to the recipient which, in turn, may deprive the person of a dignified death.^5 6^ Injuries may include rib fractures, sternal fractures, as well as cardiac, pulmonary or intra-abdominal organ injuries.^7^,^9^ Given the potential for serious consequences, it is necessary to ensure that the decision-making process reflects the person’s wishes and is effective and appropriate. The initiation of the do not attempt cardiopulmonary resuscitation (DNACPR) decision-making process may occur when there is clinical evidence that CPR would be futile, that resulting harm would outweigh potential benefits or if a patient refuses CPR treatment. DNACPR decisions do not however involve decisions about acute life-saving treatments.^10^ In addition to clinical considerations, the decision-making process is underpinned by human rights and respect for decision-making capacity. From a human rights perspective, rights such as the right to life, the right to be free from inhuman and degrading treatment, and the right to autonomy should guide the DNACPR decision-making process. In relation to mental capacity, one must assume that all adults can make their own decisions unless a capacity assessment shows otherwise. Based on this framework, national guidelines and policies have been developed across countries which describe the context, setting and process for informed decision-making for DNACPR decisions. However, evidence on the decision-making process is lacking and variations exist across cultures, countries and health conditions. In this respect, the focus of this paper is to conduct a scoping review to explore the evidence of the process of DNACPR decision-making.

## Methods

A scoping review was chosen to present a broad understanding of the area and to map the available literature relevant to the research questions in a manner not restricted by study quality or design.^11^ Scoping reviews plot publications by classifying constituents of the literature, such as design of the study, population, setting, intervention, theoretical or conceptual framework, aspects of importance and results. This leads to an understanding of the extent and gravity of the literature. Within this scoping review, the methodological framework by Arksey and O'Malley^11^ was used. This involved a six-step process: (a) identifying the research question; (b) identifying relevant studies; (c) study selection; (d) charting the data; (e) collating, summarising and reporting the results and (f) consulting with stakeholders to inform and/or validate study findings. Results are conveyed by means of a narrative in addition to tables.^11 12^

### Identifying the research question

To meet the aim of this review, the authors addressed the following questions: (a) timing of decision-making, (b) evidence of involvement, (c) evidence of discussion, (d) evidence of decision documented, (e) communication and adherence to decision and (f) recommendations from the literature.

### Identifying relevant studies

A wide range of search terms were used to identify the breadth of literature. This process included the use of the integrated database thesaurus, open search terms and Boolean operators. Searches were completed using thesaurus terms search, title or abstract searches, and the subsequent combination of search strings (table 1). Eight electronic databases were searched: Academic Search Complete, CINAHL, Cochrane, EMBASE, MEDLINE, PsycINFO, Scopus and Web of Science.

**Table 1:** Search terms: Medline

S1	(MH ‘decision making’) OR (MH ‘clinical decision-making’) OR (MH ‘decision making, organisational’) OR (MH ‘decision making, shared’)
S2	TI (implementation or evidence based practice) OR AB (implementation OR evidence based practice)
S3	TI (do not attempt resuscitation OR do not attempt cardiopulmonary resuscitation OR do not resuscitate OR not for resuscitation OR resuscitation order OR cardiopulmonary resuscitation OR resuscitation OR resus OR CPR OR DNACPR OR DNAR OR resuscitation plan OR DNR order OR NFR OR DRN OR allow natural death)ORAB (do not attempt resuscitation OR do not attempt cardiopulmonary resuscitation OR do not resuscitate OR not for resuscitation OR resuscitation order OR cardiopulmonary resuscitation OR resuscitation OR resus OR CPR OR DNACPR OR DNAR OR resuscitation plan OR DNR order OR NFR OR DRN OR allow natural death)
S4	(MM ‘Resuscitation Orders’) OR (MM ‘Resuscitation+’) OR (MM ‘Cardiopulmonary Resuscitation+’)
S5	S1 OR S2
S6	S3 OR S4
S7	S5 AND S6

AB, abstract search; CPR, cardiopulmonary resuscitation; DNACPR, do not attempt cardiopulmonary resuscitation; DNAR, do not attempt resuscitation; DNR, do not resuscitation; DNR, do not resuscitation; MH, Search the exact MeSH subject heading; searches both major and minor headings; MM, Searches the exact MeSH subject heading; searches just for major headings; NFR, not for resuscitation; TI, title search.

### Study selection

**Figure 1:**
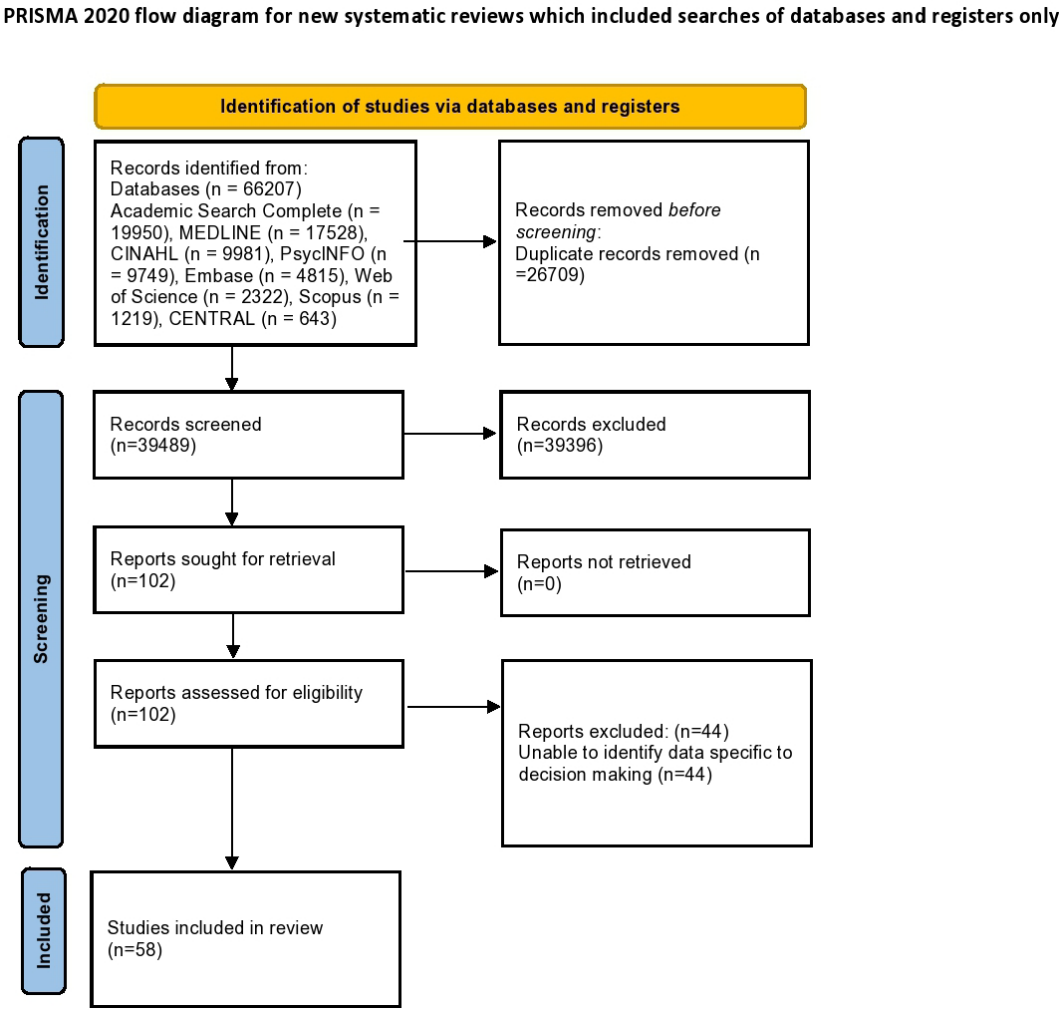
Preferred Reporting Items for Systematic Reviews and Meta-Analyses flow diagram.

Search results (n=66 207) were uploaded to Covidence where duplicates were removed (n=26 709), and screening was conducted against the inclusion and exclusion criteria (table 2). The selection process first reviewed titles/abstracts (n=39 498) followed by a full-text review (n=102). The screening process was conducted by paired reviewers and the search process is shown in a Preferred Reporting Items for Systematic Reviews and Meta-Analyses (PRISMA) flow diagram^13^ in figure 1 and the review was reported in line with the PRISMA extension for Scoping Reviews Checklist.^14^ Full-text review resulted in 44 papers being excluded and 58 being included in this review.

**Table 2:** Inclusion and exclusion criteria

Inclusion criteria	Exclusion criteria
All types of study to be included.Participants to include any healthcare professional.Participants to include patients/surrogate decision-makers.Intervention—any type of intervention for decision-making or implementation.Any type of outcome to be included.Published from 1 January 2013 to 6 April 2023.	Must report decision-making of DNACPR orders.Abstract/conference proceedings, editorials, letters, discussion papers, opinion pieces or commentaries, individual case studies.Pre 2013 publications.Non-English-language publications.

DNACPR, do not attempt cardiopulmonary resuscitation.

### Charting the data

A narrative outline and tables are used to present the data from this review. Data were charted into a data extraction table (online supplemental table 1) addressing the key questions identified to meet the aim of the review along with author, year, title, country, aim of study, methodology and limitations. To assist in plotting the data, a content analysis process was conducted by paired reviewers following the steps of Colorafi and Evans^15^: (1) create a coding framework, (2) add codes and memos, (3) apply the first level of coding, (4) categorise codes and applying the second level of coding, (5) revise and redefine codes, (6) add memos, (7) visualise data and (8) represent the data.

### Collating, summarising and reporting the results

The findings of this review were charted, collated, summarised and reported based on the researchers’ analysis of the papers that met the review criteria. This was a flexible approach to analysis that allowed for ordering, coding, categorising and summarising of data to be presented under the six questions identified to meet the aim of the review and make recommendations to inform health service policy and research.

### Consulting with stakeholders to inform or validate study findings

The importance of integrating expert consultation within the scoping review process is emphasised, but often this step is overlooked.^12 16^ An expert group from the Health Service Executive (HSE) in Ireland provided consultation into the process of searching (terms), interpretation (results), presentation (findings) and recommendations (policy/education/research) for this scoping review and acted as a research steering group.

## Results

### Study characteristics

Of the 58 studies that were included in this scoping review, 36 were quantitative, 19 qualitative and 3 mixed methods in design (table 3). The range of studies spanned across 20 individual countries, and one study was conducted in the Middle East region and one in both the USA and the UK (table 3). The highest number of studies were conducted in the UK and in Taiwan (n=9, 15.5%), with multiple studies in most countries (n=46, 79.3%) and only one study in 12 countries (20.7%). In one study, Gibbs *et al*^17^ spanned across 43 countries in total and was counted separately.

**Table 3:** Study characteristics

Type of study	Study
Quantitative (n=36)	16 1820
Qualitative (n=19)	1923 36 41 43 49 50 52 54 57 62 63 66 67 69
Mixed methods (n=3)	27 47 71

Country of origin	Study
United Kingdom (n=9).	18 24 25 41 47 49 50 58 71
Taiwan (n=9).	2133 36 61 63 65 68
Switzerland (n=6).	19 23 26 29 44 54
United States of America (n=6).	51 59 62 66 70 73
Saudi Aribia (n=5).	20 48 56 64 72
Netherlands (n=3).	22 39 45
Australia (n=2)	40 74
Canada (n=2).	32 67
Sweden (n=2).	43 46
Bahrain (n=1).	65
China (n=1).	69
Denmark (n=1).	42
Hawaii (n=1).	55
Iran (n=1).	53
Ireland (n=1).	27
Israel (n=1).	58
Japan (n=1).	28
Jordan (n=1)	60
Korea (n=1)	30
Middle East (n=1)	31
New Zealand (n=1)	57
USA and UK combined (n=1)	52
Argentina, Australia, Austria, Barbados, Belgium, Brazil, Brunei, Canada, Colombia, Cuba, Denmark, France, Germany, Greece, Hong Kong, Hungary, Iceland, India, Ireland, Israel, Italy, Japan, Lebanon, Malaysia, Malta, Netherlands, New Zealand, Norway, Pakistan, Poland, Puerto Rico, Saudi Arabia, Singapore, South Africa, South Korea, Spain, Sri Lanka, Sweden, Switzerland, Taiwan, UAE, Uganda, USA (n=1)	17

### Timing of decision-making

It is acknowledged that there is a need to make DNACPR decisions, as soon as possible in a patient’s admission,^18^ as part of a timely process^19^ and for this to be commenced as early as possible after a diagnosis of an incurable disease.^20^ There is a lack of clarity as to when the right time is for decisions to be made within the admission-to-discharge journey. Discussions were held in the emergency department,^21^ admission period,^22 23^ after admission,^24^ within 48 hours of admission,^25^ within 72 hours of admission^26^ or following admission secondary to their life-limiting illness.^27^ More broadly, Abe *et al*,^28^ Becker *et al*^29^ and Choi *et al*^30^ identify DNACPR decisions within the hospitalisation period, while Abe *et al*^28^ identified 9.2% of discussions as occurring during an outpatient visit. Typically, end-of-life issues were identified as being discussed prior to becoming severely ill,^31^ being informed of a poor prognosis and/or referred for palliative care,^32^ having 4–6 months to live,^33^ disease progressed to terminal stage,^34^ last week of life,^35^ health deteriorated,^36^,^39^ person moving to a residential setting,^39^ at a care plan meeting^39^ and inpatient discharge.^38^

Physicians have a direct influence on the timing of DNACPR discussions,^33^ and the ideal timing of a DNACPR discussion may differ between patients.^40^ Timing was influenced by patients’ perceived understanding that they are, or will be, dying.^41^ However, the attitudes of patients and physicians may differ regarding both the decision-making authority and the timing of the DNACPR decision.^42^ Generally, patients did not consider DNACPR or found it hard to think about when their physical condition was good.^36^ However, conversations need to be held when the patient is healthy,^34^ they need to be ongoing so a process occurs over time rather than as a single event,^43^ yet standardising the timing and format of DNACPR discussion to fit hospital culture may distress patients.^40^

### Evidence of involvement

Patient involvement in the decision-making process was reported between 33% and 55% of the time; 33%,^28^ 48%,^22^ 52.8%^44^ and 55.8%.^45^ Almost half of the respondents in one study reported that it was not likely that the patient would be involved in the decision on DNACPR^46^; while in another study 66% of decisions were made by the medical team without asking the patient or those close to the patient.^26^ DNACPR decisions were dominated by the belief that patient inclusion is often pointless.^19^ Patients’ mental or cognitive function was seen as the most important barrier to their involvement in resuscitation decisions.^47^ Other reasons for lack of involvement were down to physicians’ abilities to communicate with patients/surrogate decision-makers about DNACPR decision-making; 15% of physicians prefer to discuss the topic only if the patients/surrogate decision-makers bring it up.^33^ Only a minority of participants (9.3% of junior paediatricians vs 12.5% of senior paediatrician) reported that they would be comfortable discussing DNACPR with parents.^48^ General practitioners felt they should discuss the decision with the patient, but they have anxieties around this.^41^ Patients who were cared for by a family medical physician prior to death at last hospitalisation and those who had received hospice palliative care were more likely to have signed a DNACPR letter of intent.^35^ Getting to know the patient and developing a rapport are crucial factors in feeling confident to have the DNACPR conversation.^49^

Doctors acknowledged the need to involve patients and/or those close to the patient in resuscitation decisions, but there were occasions when this did not occur. Doctors hold varying perspectives about what involvement requires.^50^ General practitioners vary widely in how much they guide patients and those close to the patient in decision-making^41^; 69% of resident physicians who were unwilling to offer a DNACPR recommendation stated that the need for patient autonomy prevented them from providing guidance.^51^ Nonetheless, many CPR/DNACPR conversations are characterised by a nudging communicative approach where the physician pushes patients to their recommendation, despite the belief that conversations should be as neutral as possible.^19^ Discussions with the patient or those close to the patient from a goal-of-care perspective took place with a focus on comfort care without explicitly mentioning resuscitation.^52^

### Evidence of discussion

There was evidence of discussion with either the patient or those close to the patient in 68.7% of cases.^25^ For most documented DNACPR conversations, it was unclear however who had initiated the discussion and how the decisional power was shared.^42^ Practice was influenced by observation of other health professionals communicating DNACPR decisions with patients.^49^ Most patients and caregivers had already thought about DNACPR in anticipation or preparation of having this discussion with the healthcare provider^32^; with only 37.5% of them having DNACPR knowledge. DNACPR knowledge had a significant relationship with age and educational level^53^; 57% of practitioners reported that providing information to the patient was important and 21% stated that this was likely to happen.^46^ There were differences between nurses and physicians, regarding participation by and information to patients and those close to the patient.^46^ Among practitioners, 46.7% of respondents reported they were likely, or very likely, to share prognostic information.^51^ Where the risks and outcomes of CPR were discussed, they were provoked by the patient’s misunderstanding of a question or uncertainty.^54^ Patients’ desires related to the seriousness of an imagined or future medical state^32^; culture and religion are factors that can influence decision making,^17 55 56^ 76.2% of physicians rated the influence of religion on code status decisions and 82.9% of physicians rated the influence of culture.^29^ Religion plays a role in making DNR decisions for 58.3%^31^ and the importance of comfort during dying was a priority for 45.3%.^31^ A clear distinction was evident between countries in which there is a culture of patient autonomy and those where the personal rights of the individual are viewed as less important. Similarly, religion was a clear factor in the decision in countries with strong religious majorities.^17 55 56^

Doctors described feeling under pressure to have discussions about resuscitation with all patients soon after their admission. They acknowledged what a sensitive topic resuscitation could be and the difficulty in having such discussions with patients and those close to the patient whom they had never met before.^50^ Patient characteristics can inhibit conversations; these include engaging with younger patients, anxious patients, those who find it difficult to discuss their future and end-of-life issues, patients with an unrealistic view of their disease and those who have not come to terms with their prognosis.^38^ Factors affecting DNACPR conversations included patient acceptance, how well a rapport had been established and finding a time when there were less interruptions.^49^ The setting for the discussions was felt to be a barrier due to issues such as lack of privacy and the difficulty in finding appropriate time.^47^ Practitioners may not discuss resuscitation decisions if they believe that it would be distressing or the person lacks capacity^47^; general practitioners feel they should discuss the decision with patients who have capacity but are fearful of removing hope.^41^ Patients themselves favoured having time to discuss their decision with medical staff and family and recognised that this decision could change over time and because of altered circumstances, according to the progression of their disease.^40^ Factors that influenced the decision were the wish for a natural death, advanced age and a realistic awareness about the consequences of resuscitation.^57^

### Evidence of decision documented

Evidence of decisions being documented varied from 19% to 93.9% in the studies that presented percentages (93.9%,^28^ 92.3%,^25^ 91.2%,^45^ 70.9%,^58^ 60%,^42^ 57%–64%,^59^ 21.2%^44^ and 19%^22^). Coleman *et al*^24^ reported DNACPR decisions recorded and Khalaileh^60^ identified DNACPR orders in nursing notes. However, medical orders respecting DNACPR were not documented in 71.9% of patients' records.^53^ There was a 23.7% disagreement between preference given and documented code status in the medical electronic chart.^29^ For 40% of patients with an advance directive, their preference did not match the documented code status.^29^ Signing of forms varied, with 56.35% personally signing,^35^ 23% of the surrogates signing,^21^ 11.3% signed by either residents or family surrogates.^61^ No patient with a DNACPR order provided written informed consent by themselves.^30^ Healthcare professionals thought it important that the care team would be informed of the decision and that the decision would be clearly documented^46^ and 87% worked in a hospital where there was a method for communicating decisions to their medical colleagues.^17^ Physicians asked those close to the patient to sign the DNACPR order, to confirm that they had seen or approved it,^39^ but mostly felt it was their professional decision and did not want to burden those close to the patient.^39^ 81% preferred a coding system to a written statement^60^ and it was recommended that the form for recording the instructions on DNACPR should be flexible enough to accommodate a spectrum of possibilities.^40^

### Communication and adherence to decision

Communication and adherence to the DNACPR decision were influenced by understanding and awareness, the decision maker(s), policy and guidelines, and the options and resources available. There is a need for clear direct communication and validation of whether the patient and the caregiver understand the implications of DNACPR^32 62^; where an awareness video was used people said they did not want intubation.^59^ Training and background of healthcare professionals were significant factors affecting the interpretation of the term no code DNACPR.^31^ When physicians were determining the DNACPR status for a patient, conflicts were reported at a higher rate (62.4%).^29^ Within the specific population of intellectual disability, choking is seen as an accident and should be treated as a non-natural death, implying that the person should be resuscitated even if a DNACPR order had been issued [6139]. When the patient was absent from the DNACPR discussion, decisions were made by those close to the patient based on discussion with the physician^28^ or by a senior doctor, with a consultant countersignature.^25^ However, if patients and those close to the patient would not accept a DNACPR order, CPR was given till the patient’s death based on the applicable law^36^; where patients expressed their agreement clearly, the DNACPR order was adhered to in order to respect their autonomy.^63^ Of note was that 50% of physicians stated that it is unimportant to review the DNACPR order periodically and that there is no need to undo it for any reason.^64^ There was a strong emphasis on the need for a clear DNACPR policy with 98%,^65^ 94%,^17^ 74%^60^ of their samples reporting the need for policy and that there should be legislation regarding DNACPR.^65^ It is recognised that there are substantive differences in the design of hospital code status options which may contribute to known variability in end-of-life care and treatment intensity among USA hospitals^66^ and data supports the idea that clinicians followed the recommendations to initiate treatment limitation conversations.^24^ However, ethical attitudes toward DNACPR decision-making reflect hospital policy regarding prioritisation of autonomy over best interests,^52^ and resources such as ethics consultation should be used.^67^

### Recommendations from the literature

The recommendations can be categorised under headings to include the content and timing of the DNACPR discussion, the influence of healthcare professionals, the role of patient autonomy, the place of the family in decision-making, training and professional development, and procedural elements. On the issue of timing, the early initiation of DNACPR discussions was recommended by Abe *et al*,^28^ Cheng *et al*,^21^ Coleman *et al*^24^ and Einstein *et al*.^51^ Proactive discussion^35^ can allow the person to fully participate in the decision^30^; it also ensures that familiar healthcare professionals are involved in the conversation.^49^ To be effective, these discussions require protected time^32^ and should be held in an appropriate environment.^19 23^ For decisions involving children, Liu *et al*^37^ highlight that doctors and nurses need to sensitively explain the pros and cons of signing a DNACPR form and patiently wait for parents to assimilate the information so as not to rush to a final decision. Moaed *et al*^58^ noted that clarification of DNACPR status allows timely planning of the best treatment for actively dying children, thereby facilitating the avoidance of futile and unnecessary medical treatment. This may also help to reduce the psychological stress imposed on medical and other staff treating the dying child.^58^

Healthcare professionals have a significant role in initiating^63^ and facilitating discussions between caregivers, patients and those close to the patient.^63^ While there is faith placed in the doctor’s own view,^32^ this may be shaped by their views on autonomy.^28 44 60^ Bedulli *et al*^19^ indicated that patient’s wishes would need to be a priority. This reflects the right of self-determination^43^ and needs to be protected.^68^ Decisions are not to be made in the abstract, they require that the patient be appropriately informed.^45 54^ Ramages and Cheung^57^ highlight the importance of educating people as to the potential outcomes of resuscitation, and exploring and documenting their reasoning when discussing resuscitation preferences so that their wishes can be respected. The need for patient involvement is further underlined by Hadley^49^; DNACPR decisions that do not include agreement from patients or those close to the patient and operate instead on multidisciplinary team agreement is likely to be unsuccessful and inappropriate. Familiarity with DNACPR decisions may not be consistent across all multidisciplinary team members,^53^ but team meetings can build confidence and support difficult end-of-life decisions.^41^ Although there is concern about the impact of these discussions on the patient, Low *et al*^38^ demonstrated that most patients deal with these discussions much more positively than staff anticipate.

In addition to providing information to the patient, Alsaati *et al*,^20^ Alwazzeh *et al*^64^ and Ding *et al*^69^ recommend that healthcare professionals also provide an explanation of DNACPR decisions to those close to the patient. This can help avoid misunderstandings, avoid stress on those close to the patient and create a better atmosphere for communication. In a UK-based study, it was noted that resuscitation decisions are often made by the healthcare professionals in charge of the patient’s care, with the views of close relatives considered.^47^ In contrast, a Bahrain-based study conducted by Ismail *et al*^65^ found that involving those close to the patient in DNACPR decisions was socially and culturally unacceptable from an Islamic perspective. Instead, it was recommended that doctors make the decision as a team and keep those close to the patient informed.^36^

Several papers highlighted the need for structure surrounding the discussion of the DNACPR decision. For instance, Ahmed *et al*^32^ identified a need for an organised evidence-based approach to guide difficult discussions. Binder *et al*^70^ set out that framing these discussions as an example of informed consent may be an effective strategy to educate people and to improve the quality of the discussion. The need for greater awareness of the roles in DNACPR decision-making and the timing of the decision have been highlighted by Saltbæk *et al*,^42^ while Taubert *et al*^71^ highlight that discussions about CPR and DNACPR need to be more routine and meaningful.

Communication difficulties have been shown to be a barrier to DNACPR discussion.^19^ On this point, Sterie *et al*^23^ highlight that there is a need for communication training regarding the involvement of patients in conversations about goals of care. Communication issues may also be overcome using patient videos which can facilitate discussion of resuscitation options.^29^ Becker *et al*^29^ also recommended the use of communication workshops focusing on exploring patients’ values and goals of care. The need to improve communication was a recurring topic across the papers examined. Aljohaney and Bawazir^72^ recommended an evidence-based curriculum providing instruction for improving discussion and Chen *et al,*^33^ ^35^ Einstein *et al*,^51^ El Sayed *et al*^67^ and Fan and Hsieh^36^ all highlight the need for training to enhance communication skills.

Communication issues may be overcome through the use of patient videos which can facilitate discussion of resuscitation options.^29^ El-Jawahri *et al*^59^ recommended a video decision support tool. This tool can inform patients’ preferences regarding CPR and intubation and increase physician–patient conversations on these topics in the inpatient setting.^35^ Videos, apps and websites were identified by Taubert *et al*^71^ as a way of facilitating understanding. A prognosis-focused discussion aid brochure has been proposed to improve patient-provider dialogue, with the potential to also improve prognostic awareness.^73^ Sritharan *et al*^74^ and Chen *et al*^33^ both draw attention to the need for education and training on legal and ethical issues for healthcare professionals. This training could clarify matters relating to resuscitation documentation, support decision-making and promote the early discussion of a patient’s care goals.^74^ On the issue of administration and procedure, Khalaileh^60^ recommended that a standard DNACPR form be kept in the patient’s medical record. It has also been proposed that the documentation of a DNACPR decision should be flexible, allowing for the doctor to provide a written narrative and to summarise the patient’s understanding of treatment choices and prognosis.^40^ The use of electronic documentation for DNACPR was addressed by Harrington *et al*^25^ who noted that this is complex and that effective implementation requires the construction of systems that enable high-quality record keeping and timely communication.

## Discussion

The concept of autonomy is central to many of the clinical, legal and ethical challenges, which arise in the context of end-of-life decisions.^75 76^ This is not a homogeneous concept as the ethical basis for autonomy is varied, and there are a multitude of perspectives on autonomy.^77^ It is therefore not surprising that the literature demonstrated considerable divergence on the exercise of patient autonomy and related components. The findings from this review highlight that both timing and equality in the relationship between medical practitioners and their patients/those close to the patient are essential.^78^ It is this relationship that enables an open conversation and dialogue to occur thereby facilitating the DNACPR decision-making process.^35^ Medical practitioners are expected to share information, knowledge and clinical decisions with their patients to help patients and those close to the patient to come to an informed decision. This process should encompass discussing the pros, cons and options in a way that will not provoke anxiety or conflict between the relevant parties. This demonstrates positive regard for patient choices and, accordingly, emphasises respect for patient autonomy as a revered principle in contemporary bioethics.^77 79^

The review drew attention to how DNACPR decision-making can be affected by cultural considerations and the ethnicity of decision-makers, along with other factors, such as religion, legal concerns, economic issues, availability of resources/technologies and perceptions of patient’s quality of life.^80^ Variations can be found across countries and, for some, involving the patient and those close to the patient in decision-making is socially and culturally unacceptable. In these countries, doctors make the decision and keep those involved informed. Appropriate and timely communication with the patient, those close to the patient or surrogate decision-maker is vital in discussing a patient’s diagnosis, prognosis, preferences and quality of life in making the right decisions for the individual patient.^81 82^ In discussions, practitioners must consider the patient’s knowledge, values and preferences in reaching a consensus.^35 83^ Such an approach reinforces the move towards personalised medicine, where the care provider–patient relationship has changed from medical paternalism to an autonomy-based relationship, in which patient participation in decision-making is a responsibility.^84^ What is evident is that variations in understanding, application and process exist and that conversations remain an important part of decision-making. These conversations need to be framed using a person-centred approach, conversing with individuals about what matters most to them in their care and identifying their ideas and wishes.^85^ A recent development has occurred in Wales where a competency framework is being developed by health boards, trusts and partner organisations to support healthcare professionals in receiving appropriate training and preparation to discuss, complete and sign DNACPR forms.^86^ In addition, a quality improvement project looking at improving DNACPR documentation in Wales showed that the introduction of a new national form resulted in clearer documentation of discussions held with patients and those close to the patient and documented reasons why and when conversations had not taken place.^87^

### Strengths and limitations

While this review uses a methodological framework and reporting guideline, no quality appraisal was conducted, as the focus of this review was to update and map the evidence. Thus, this paper only offers a descriptive account of available information, and greater patient and public involvement opportunities for engagement may have strengthened the review through stakeholder involvement. Eight databases were searched, which can be seen as both a strength and a limitation in terms of the inclusion/exclusion of low-income and middle-income countries and the exclusion of secondary data.

## Conclusion

The scoping review set out to highlight barriers and facilitators to DNACPR decision-making, processes and implementation. As part of this, the review concentrated on the timing of the decision, evidence of involvement, the nature of the discussion, documentation, communication and adherence to the DNACPR decision. The review highlighted that the issues arising under each of these headings are relatively consistent across jurisdictions. Moreover, these issues have proven consistent over time as demonstrated by a systematic review conducted by Mockford *et al*^88^ which reviewed data from January 2003 to July 2013. It may therefore be expected that these challenges will endure in a given jurisdiction unless substantial changes are made across the DNACPR decision-making framework. This does not necessarily require wholesale change but should include an effort to identify, support and promote examples of existing good practices.

Barriers arising in DNACPR decision-making related to timing and time pressure, patient and family input, weaknesses in communication, as well as conflicts and ethical uncertainty. By contrast, the facilitators included ongoing conversation along with the time to discuss the decision, the documentation of the DNACPR decision, flexibility in recording instructions on the DNACPR form, resources to support communication and the existence of clear DNACPR policy. It is unfortunate that the barriers were more prevalent than the facilitators of good practice; however, this did allow for multiple recommendations to be advanced. These are related to issues such as the content and timing of the DNACPR discussion, the influence of healthcare professionals, respect for patient autonomy, the place of the family in decision-making, training and professional development and procedural elements. Overall, the review underlined the complexity of DNACPR decision-making and how it is a process shaped by multiple factors including law and policy, resource investment, healthcare professionals, those close to the patient and, of central importance, the patient.

## Supplementary material

10.1136/spcare-2023-004573 online supplemental file 1

## Data Availability

All data relevant to the study are included in the article or uploaded as supplementary information.
