# Supplementary material for: Do not attempt cardiopulmonary resuscitation decision-making process: scoping review
Source: BMJ Support Palliat Care. 2024 Mar 22;15(3):e004573. doi: 10.1136/spcare-2023-004573 (PMC12128779; doi:10.1136/spcare-2023-004573)
Supplement: online supplemental file 1 [file spcare-15-3-s001.pdf]

Supplementary table 1: Data extraction table

**Author/s, Year, Title, Country**	**Aim of study**	**Methods and methodology**	**Decision making process**					**Recommendations / Implications**	**Limitations**
			Timing of decision-making	Evidence of involvement	Evidence of discussion	Evidence of decision documented	Communication and adherence to decision		
[28] Abe et al. (2021) Patient participation and associated factors in the discussions on do-not-attempt resuscitation and end-of-life disclosure: a retrospective chart review study.<br><br>Japan	To investigate patients' participation rate in the discussion of CPR / DNAR.	Retrospective chart review study. Hospitalised patients who died. University hospital in Japan. Patients' medical charts, April 2018 to March 2019. Statistical analysis. Ethical approval.	For 305 patients (90.8%), the discussions took place while hospitalised and for 31 patients (9.2%), during an outpatient visit.	Of the 336 patients who had DNAR discussions, 112 patients (33.3%) participated in the discussions.	21 patients (5.9%) and their families had no opportunity to discuss DNAR (unexpected death). Reason for patients' not participating in the discussion was the patient's decreased consciousness (n=119: 48.6%), doctors' judgment (n=107: 43.7%), and family requests (n=11: 4.5%).	Discussions on DNAR were evident in 336 out of the 358 patients (93.9%).	When the patient was absence from the DNAR discussion, DNAR decisions were made by patients' family based on discussion with physician.	Discussions should be initiated earlier. Discussions are influenced by patient's preferences, readiness, and medical condition. Patients' participation in discussion can be affected by the judgment of the physician regarding autonomy.	Retrospective chart review only. Quality of reports dependent on individual recording. One hospital only. Over representation of males in sample.

[32] Ahmed et al. (2015)<br>How, when and where to discuss do not resuscitate: A prospective study to compare the perceptions and preferences of patients, caregivers, and health care providers in a multidisciplinary lung cancer clinic.<br><br>Canada	To explore the perceptions and preferences of patients, their family caregivers and their health care providers in response to DNR discussions.	Prospective descriptive study.<br>10 patients, 9 caregivers, 10 healthcare providers.<br>Tertiary care cancer care in Canada.<br>Questionnaire and interviews.<br>Timeline not identified.<br>Statistical analysis, content analysis and constant comparison techniques.<br>Ethical approval.	Patients and caregivers felt the most appropriate time to discuss DNR was either when patients were informed of poor prognosis and/or referred for palliative care.	Not within the focus of the study.	Patients' desires related to the seriousness of an imagined or future medical state 'don't want to be put on machines.<br>Most patients and caregivers had already thought about DNR in anticipation or preparation of having this discussion with the healthcare provider.<br>Healthcare providers try to balance keeping the hope going as there is a huge psychologica l impact when we need to talk about DNR.	Not within the focus of the study.	Clear, direct communication and validation of whether the patient and caregiver understood the implications of DNR.	Most patients expressed 'faith' in their doctor's discretion and the doctor's responsibility to identify time and opportunity to initiate the DNR discussion.<br>Caregivers placed their 'faith in the health system'.<br>Need for more or protected time to engage in difficult DNR discussions, an organised, evidence-based approach to guide difficult DNR discussions, the involvement of other healthcare disciplines to meet the patients' multi-faceted needs and training in an appropriate, personalised approach to DNR discussions.	Small scale study.<br>Reasons for low understandin g of DNR among patients not explored.

[48] Aljethaily et al. (2020) Pediatricians perceptions toward do not resuscitate: A survey in Saudi Arabia and literature review.<br><br>Saudi Aribia.	To explore the pediatricians attitudes and perceptions toward do-not-resuscitate (DNR) orders in a specific region of the world not fully explored before.	Cross-sectional study.<br>203 pediatricians.<br>Three public hospitals in Riyadh.<br>Survey conducted between March and May 2018.<br>Statistical analysis.<br>Ethical approval.	Not within the focus of the study.	Only a minority of participants (9.3% of junior pediatricians vs 12.5% of senior pediatrician) reported that they would be comfortable discussing DNR with parents.	Not within the focus of the study.	Not within the focus of the study.	Not within the focus of the study.	Factors may hinder DNR implementation, such as doubts about being legally protected, consistency with Islamic sharia, unclear policies/procedures, and lack of training/orientation on DNR issues.<br>DNR policies need to include patients/parents as decision-makers and place them at the center of the discussion.	Study in a specific region where Islamic sharia is a key influence on approach to decision-making.
[72] Aljohaney and Bawazir. (2015) Internal medicine residents' perspectives and practice about do not resuscitate orders: survey analysis in the western region of Saudi Arabia.<br><br>Saudi Arabia.	To analyze the perceptions and practices of internal medicine residents regarding the implementation of do not resuscitate orders.	Cross-sectional study.<br>156 medical residents.<br>Western region of Saudi Arabia (Jeddah, Makah, Medinah, and Taif).<br>Survey April 2013.<br>Statistical analysis.<br>Ethical approval.	Not within the focus of the study.	Islamic sharia states the decision is clinical and family members are not qualified to be involved in the decision-making.	Not within the focus of the study.	Not within the focus of the study.	Not within the focus of the study.	An evidence-based curriculum providing instruction for improving discussions regarding DNR orders would improve physician confidence and effectiveness in caring for critically ill patients.	Study in a specific region where Islamic sharia is a key influence on approach to decision-making.

[20o] Alsaati et al. (2020) The concept of do not resuscitate for the families of the patients at King Abdul-Aziz University Hospital.<br><br>Saudi Arabia.	This study is aimed to assess the knowledge of the patients' relatives about DNR concept and their opinion about the DNR decision-making.	Cross-sectional study.<br>420 family members.<br>Emergency department of a University Hospital in Saudi Arabia.<br>Interviewed using a validated questionnaire during 2016.<br>Statistical analysis.<br>Ethical approval.	59% of participants thought that the DNR discussion should be initiated as early as possible; soon after the patient is diagnosed with an incurable disease.	The current understanding of DNR is that the involvement of the patient in the DNR decision-making process is unimportant and unnecessary.	The decision is discussed when made but neither the patient nor the family are involved in the decision-making.	Not within the focus of this study	Not within the focus of this study	A lack of understanding of the concept of DNR decision-making. Health-care providers should provide a greater explanation of DNR orders to families to avoid misunderstandings and avoid any stress they might encounter in such situations.	Specific region where Islamic sharia is a key influence on approach to decision-making.
[64] Alwazzeh et al. (2023) Knowledge gaps, attitudes, and practices regarding end-of-life medical care among physicians in an academic medical center.<br><br>Saudi Arabia.	To explore physicians' knowledge, attitude, and practice regarding end-of-life medical care and DNR.	Cross-sectional study.<br>264 physicians.<br>University hospital in Saudi Arabia.<br>Questionnaire April 2019 to March 2020<br>Statistical analysis.<br>Ethical approval.	Not within the focus of this study.	Not within the focus of this study.	Not within the focus of this study.	Not within the focus of this study.	50% of the participants stated that it is unimportant to review the DNR order periodically and that there is no need to undo it for any reason.	Consideration needs to be given to patients and family's right to be informed and involved in the decision-making process.<br>The presence of different DNR policies, lack of physicians' knowledge, and negative attitudes regarding DNR emphasise a need to update DNR policies.	Specific region where Islamic sharia is a key influence on approach to decision-making.

[55] Ariyoshi et al. (2018) Wanted and unwanted care: The double-edged sword of partial do-not-resuscitate orders.<br><br>Hawaii.	To investigate the effect of partial DNRs on physicians' willingness to perform cardiopulmonary resuscitation (CPR) and nonarrest procedures.	Cross-sectional study.<br>275 hospitalists attending physicians.<br>A tertiary-care academic medical center.<br>Questionnaire October 2015 to March 2016.<br>Statistical analysis.<br>Ethical approval.	Not within the focus of this study.	Not within the focus of this study.	Not within the focus of this study.	Not within the focus of this study.	Not within the focus of this study.	Findings suggest an on- going need to develop and validate better means of incorporating patients' medically achievable goals of care into orders that more faithfully guide care for both prearrest and arrest situations.	A single-center survey.
[66] Batten et al. (2021) Variation in the design of Do Not Resuscitate orders and other code status options: A multi-institutional qualitative study.<br><br>United States of America.	To explore design differences in hospital code status options and the perceived impacts of these differences.	Qualitative design not specified.<br>30 physicians.<br>7 USA hospitals.<br>Interviews, policy documents and records of ordering menus, time period not specified.<br>Triangulation, conventional content analysis and systematic framework for evaluating the design of code status options.	Not within the focus of this study	Not within the focus of this study.	Not within the focus of this study.	Not within the focus of this study.	There are substantive differences in the design of hospital code status options which may contribute to known variability in end-of-life care and treatment intensity among USA hospitals.	The study assessed the perceived impacts of the design of code status options but did not link design differences to observed differences in communication, decision-making, clinical outcomes or overall treatment intensity.	Included physicians only and other healthcare professionals not included.

		Ethical approval.							
[29] Becker et al. (2020) Code status discussions in medical inpatients: results of a survey of patients and physicians.<br><br>Switzerland	To understand current practices and perceptions of code status discussions in a tertiary-care Swiss hospital.	Cross sectional study.<br>258 physicians and 145 patients.<br>A university hospital in Switzerland.<br>Interviews using a questionnaire between May and July 2018.<br>Statistical analysis.<br>Ethical approval not specified.	Code status decision made during hospitalisation.	61.4% of patients did not recall having a code status discussion during the hospital stay.<br>43.6% of medical patients compared to 22.4% of surgical patients recalled having had a discussion.<br>72.4% of physicians recalled defining a DNR status for a patient without prior discussion with the patient.	76.2% of physicians rated the influence of religion on code status decisions to be more than 50 on a 0-100 scale.<br>82.9% of physicians rated the influence of culture to be more than 50 on a 0-100 scale.	For 23.7% of patients there was a lack of agreement between the preference given in the interview and the documented code status in medical electronic chart.<br>One patients code status was not documented. For 40% of patients with advance directive, their preference did not match the code status documented in the patient record.	Physicians who recalled determining DNR status for a patient consultation reported conflicts with patients and relatives regarding code status at a higher rate (62.4%) compared to physicians who did not define DNR status without consultation (39.4%).	Code status discussion training may improve informed decision-making and patient centred care.<br>Accessibility and availability of advance directives is required.<br>Patient videos may facilitate code status discussions and overcome communication issues.<br>Communication workshops exploring patients' values and goals of care and identifying patients at risk of poor decision-making is required.<br>Hospital policy should ensure code status discussions is accommodated.	Physicians interviewed may not have been the treating physicians involved in the care of the interviewed patients.<br>Singe centre survey.<br>Orthopaedics, Cardiac and Vascular Surgery areas only included.

[19] Bedulli et al. (2023) Obstacles to patient inclusion in CPR/DNAR decisions and challenging conversations: A qualitative study with internal medicine physicians in Southern Switzerland.<br><br>Switzerland.	To explore physician reported CPR/DNAR decision-making approaches and CPR/DNAR conversations with patients hospitalised in medicine wards.	Qualitative design not specified.<br>19 resident and staff physicians.<br>Four hospitals in Switzerland.<br>Focus group interviews July 2021.<br>Thematic analysis.<br>Ethical approval.	Patients should be engaged in a timely process to make decisions on future care plans, particularly anticipating the possibility of severe conditions and eliciting what treatment outcomes patients would consider acceptable.	CPR/DNAR decisions were dominated by the belief that patient inclusion is often pointless. Mostly CPR/DNAR conversations are characterised by a nudging communicative style where physicians direct patients to their recommendation, despite the belief that conversations should be as neutral as possible.	Not within the focus of this study.	Not within the focus of this study.	Not within the focus of this study.	CPR/DNAR conversations should be held in an appropriate environment before the acute event. Communication interventions are complex interventions that need to be learned as communication difficulties are a barrier to CPR/DNAR conversations. Patient wishes need to be a priority and when decision are made the decision needs to be discussed.	A small number of participants with a low response rate.
[70] Binder et al. (2016) Uninformed consent: Do medicine residents lack the proper framework for code status discussions.	To assess the content of code status discussions as reported by residents to examine whether	Prospective, observational, single center survey study.<br>100 medical residents.<br>Medical centre USA.	Not within the focus of this study.	66% have code status discussions with most patients upon hospital admission.	Not within the focus of this study.	Not within the focus of this study.	Not within the focus of this study.	Conversations regarding CPR are insufficient in the 5 key elements of informed consent.<br>Framing code status	Single site used.<br>Recall and selection bias.

United States of America.	residents meet requirements of informed consent.	Survey Jan-Feb 2012. Statistical analysis. Exempt from institutional review board review.						discussions as examples of informed consent may be an effective strategy for educating residents and may improve the quality of discussions leading to better patient decisions.	
[43] Brännström and Jaarsma. (2015) Struggling with issues about cardiopulmonary resuscitation (CPR) for end-stage heart failure patients.<br><br>Sweden.	To describe the experiences and thoughts of members of an integrated heart failure and palliative care team concerning talking about CPR with end-stage heart failure patients.	Qualitative descriptive study.<br>7 participants (2 doctors, 3 nurses, 1 occupational therapist, 1 physiotherapist).<br>Integrated heart failure and specialised palliative home care unit in a community hospital.<br>Group interviews every three months over one year in 2011.<br>Content analysis.	Holding an ongoing conversation, which is a process over time rather than a single event.	Family members should be included in conversations with a patient.	Doubts and uncertainties exist as to whether it is right to involve patients and their relatives in decision-making.<br>Decisions should be based on medical assessment.<br>Need to take patient's decision-making capacity into account.<br>Patient have the right to participate in decision-	Not within the focus of this study.	Not within the focus of this study.	Involving patients and their relatives in the decision-making can be challenging, but patients have the right of self-determination.<br>Conversation needs to be the start of a broader end-of-life conversation.	Small sample size.<br>Low number of health care professionals and mix of professional.<br>Data for first year of the project.

		Ethical approval.			making and their wishes must be honoured.				
[18] Chang and Matthews. (2022) How is COVID-19 changing the ways doctors make end-of-life decisions. United Kingdom.	To explore how the COVID-19 has changed how doctors make end-of-life decisions, treatment escalation and views on legalisation of euthanasia and physician-assisted suicide.	Cross sectional study. 231 doctors. Working in the national health service. Questionnaire May to August 2021. Statical analysis. Ethical approval.	An increased sense of urgency among clinicians to make DNACPR decisions as early as possible in a patient's admission.	Not within the focus of this study.	Not within the focus of this study.	Not within the focus of this study.	Not within the focus of this study.	Over half of clinicians surveyed reported that they are now making more DNACPR decisions during the pandemic.	Accessible to digital media. Respondents redeployed during COVID. Focus on doctors' perceptions of their decision making rather than on the decisions themselves.
[68] Chang et al. (2020) Nurses' experiences and factors related to their attitudes regarding discussions with patients and family members about do-not-resuscitate decisions and life-sustaining treatment withdrawal: A hospital-based	To evaluate nurses' experiences and factors related to their attitudes regarding discussions of do-not-resuscitate and withdrawal of life-sustaining treatment	Cross-sectional study. 132 nurses. Tertiary hospital Taiwan. Questionnaire August to October 2018. Statistical analysis. Ethical approval.	Not within the focus of this study.	Not within the focus of this study.	More likely to discuss DNR and life-sustaining treatment with families than with patients.	Not within the focus of this study.	Not within the focus of this study.	Patients' autonomy and rights to make decisions about their DNR and life-sustaining treatment needs to be protected. Measures to facilitate DNR and life-sustaining treatment discussions with patients should be implemented.	One tertiary hospital with a well-organised hospice palliative care team.

cross-sectional study.<br><br>Taiwan.	with patients and their families.								
[35] Chen et al. (2022) Factors influencing terminal cancer patients' autonomous DNR decision: a longitudinal statutory document and clinical database study.<br><br>Taiwan.	To analyse medical records to understand the fact of terminal cancer patients' autonomous DNR decision-making in Taiwan.	Retrospective chart review study.<br>1607 patient records.<br>One tertiary public medical center in Taiwan (patients who had died).<br>Medical information system database Jan. 2017 to Dec 2018.<br>Statistical analysis.<br>Ethical approval.	Many patients had the first DNR order within their last week of life 40.81%.	Patients who were cared for by family medicine physicians prior to death at last hospitalisation and those who had received hospice palliative care were more likely to have signed DNR letter of intent.	Patients whose final attending physicians were family medicine practitioners, and those who had received hospice palliative care were more likely to have signed DNR documents in person.	56.35% personally signed DNR letter of intent. Optional note of personal DNR wish on national health insurance cards promoted via hospital subsidies for community education.	Not within the focus of this study.	Staff need further education on legal and ethical issues around patient autonomy and training on communicating end-of-life options.<br>Proactively discussing DNR decision issues with terminal cancer patients no later than when their estimated survival is close to 1 month.	Medical records do not contain clinical details.
[33] Chen et al. (2019) Are physicians on the same page about do-not-resuscitate? To examine individual physicians' influence on do-not-resuscitate decision-making: a retrospective and observational study.	To explore the influence of individual attending physicians on signing a DNR order.	Retrospective and observational study.<br>1859 patients.<br>78 surgical ICU beds in a university hospital Taiwan.<br>The medical records of patients, admitted to the	Physicians had in-fluence on the timing of signing a DNR order for patients.<br>65% of physicians caring for cancer patients tend to	Physicians' abilities to communicate with patients/surrogate decision-makers about DNR decision-making may vary.<br>15% of physicians	Physicians influence the DNR decision made.<br>Some physicians are more likely to write a DNR order for the patients and others less	Not within the focus of this study.	Not within the focus of this study.	Educational interventions may prevent misinterpretation regarding DNR orders and facilitate discussion between healthcare professionals and patients, surrogate decision-makers.	Single center study.<br>The influence of other healthcare team members on DNR decision-making was not considered.

Taiwan.		surgical intensive care units for the first time between June 2011 and December 2013. Statistical analysis. Ethical approval.	discuss end-of-life decision-making when an asymptomatic cancer patient has four to six months to live.	prefer to discuss the topic only if the patients/surrogate decision-makers bring it up.	likely to do so. Patient's admission diagnosis and severe clinical illness upon surgical ICU admission were associated with signing a DNR order.				
[21] Cheng et al. (2016) Do-not-resuscitate orders and related factors among family surrogates of patients in the emergency department. Taiwan	To explore the prevalence of DNR orders and relevant factors influencing DNR decision-making by patients' surrogates in emergency departments.	Prospective, descriptive, and correlational study. 200 surrogate decision makers. Emergency department of a medical center in Taiwan. Interviews using questionnaire between July 2011 and March 2012. Statistical analysis. Ethical approval.	Discussions were held in the emergency department.	26% of participants had discussed the DNR order with the patient. 27% of surrogates had actively discussed the DNR decision with physicians before emergency department admission.	Not within the focus of this study.	23% of the surrogates signed a DNR order for the patient, 49.5% signed after physician initiated the discussion, 72.5% signed after an open discussion among family members and 54% signed when they understood the patient's disease	Not within the focus of this study.	Early initiation of DNR discussions is suggested to improve end-of-life care.	Convenience sampling. Patients were selected from emergency department patients which limits generalisability of findings to other settings.

						could not be cured.			
[26] Chevaux et al. (2015) Patients' characteristics associated with the decision of "do not attempt cardiopulmonary resuscitation" order in a Swiss hospital.<br><br>Switzerland	To assess the prevalence and the determinants of DNACPR orders in a Swiss university hospital.	Observational cross-sectional study.<br>194 patients.<br>Medical ward of a Swiss hospital.<br>Medical record from March and May 2013.<br>Statistical analysis<br>Ethical approval.	53% of patients had a DNACPR order within 72 hours of admission.	27% of DNACPR orders were issued by the patient, 12% by relatives, and 61% by the medical team.<br>66% of DNACPR decisions were made by the medical team without asking the patient or their relatives.	Age was the strongest factor associated with a DNACPR order.<br>Oncologic disease was also important in deciding on a DNACPR order.<br>Being protestant was a factor for patient's decision but not doctors.	Not within the focus of the study.	Not within the focus of the study.	A medical reflection should occur about further measures of sensitisation and extensive formation – medical and ethical – on CPR/DNACPR determination.	Only patients in the internal medicine ward were included.<br>Study site one hospital only.
[63] Chiang et al. (2021) The concerns and experience of Decision-Making regarding Do-Not-Resuscitate Orders among caregivers in hospice palliative care.<br><br>Taiwan.	To explore the concerns and experiences regarding DNR decisions among caregivers in Taiwan.	Qualitative design not specified.<br>36 caregivers.<br>Recruited from a hospice ward, a hospice home care in a medical center, and communities in Taiwan.<br>Thematic analysis<br>Ethical approval.	Not within the focus of the study.	Family members were involved in the decision.<br>Most participants stated that medical information and physician's suggestions were the foundation of	Carers tend to accept DNR orders when they realise CPR cannot extend life or can only increase pain or other symptoms.<br>Ability to communicate with patients influences decision-making.	Not within the focus of the study.	Where patients had expressed their willingness clearly, the caregivers tended to accept the DNR order to respect patient autonomy.	Healthcare professionals can teach caregivers how to open discussion, provide support to patients, and deal with patients and their own emotional responses.<br>Healthcare professionals can facilitate communication	All patients had DNR orders.<br>Most patients were diagnosed with cancer.

				decision-making.	Caregiver struggle making the decision when the patient do not express an opinion and reject DNR decision when they don't understand the prognosis or can't accept patient's death.			between the caregivers, patients and other family members to solve disagreements. Healthcare professionals can guide patients to think of advance care planning early.	
[30] Choi et al. (2018) Impact of frailty on do-not-resuscitate orders and healthcare transitions among elderly Koreans with pneumonia.<br><br>Korea.	To identify the decisive factors associated with DNR orders in older patients with pneumonia.	Retrospective cohort study. 512 patients. University hospital in Seoul. Medical records of 431 pneumonia patients admitted between June 2014 and May 2015. Statistical analysis Ethical approval.	Only 15.1% (n=65) patients made DNR decisions within the hospitalisation period. The median time between in-hospital mortality and the DNR order was 32 hours.	All DNR decisions were determined by surrogates.	Persons with a completed DNR document tended to be older and frail, with higher rates of renal impairment and malnutrition, and had a lower microbiology detection effort. Frailty was independently associated	None of the patients with a DNR order provided written informed consent for the DNR by themselves.	Not within the focus of the study.	Efforts should be made to speak to very older adults about their health values when they are still healthy and able to understand advanced directives.	Retrospective design and unable to identify cases where proposed DNR orders were rejected. Just focused on pneumonia admissions.

					with DNR status.				
[24] Coleman et al. (2020) Bringing into focus treatment limitation and DNACPR decisions: How COVID-19 has changed practice.<br><br>United Kingdom.	To analyse electronic healthcare records documentation on treatment limitation and DNACPR decisions comparing uptake, decisions and answers to different sections of the form before and during the COVID-19 situation.	Retrospective study.<br>16,007 records.<br>Large urban teaching hospital in England.<br>Eletronic health records between January 2017 and April 2020.<br>Statistical analysis.<br>Ethical approval not specified.	DNACPR/ Treatment Escalation and Limitation form completed after admission to hospital.	The proportion of discussions that involved the patient remained similar, 95.6% 2019 95.8% during COVID-19.<br>Significant decrease in DNACPR/ Treatment Escalation and Limitation decisions being discussed with the relatives dropping from 75.4% to 50.6%.	During COVID-19 where a decision about resuscitation was recorded, the patient tended to be younger (79 pre-COVID vs 74 during COVID); less likely to have various comorbidities and less likely to have a referral to the palliative care team (16.7%/28.5 %).	Decisions recorded in DNACPR/ Treatment Escalation and Limitation forms.	Data supports the idea that clinicians followed the recommendations to initiate treatment limitation conversations with more inpatients during this period.	The study recognises that making early decisions surrounding ceilings of care has multiple benefits.<br>Necessary to minimise potential ‘wastage’ of PPE by avoiding inappropriate CPR.	There may be an absence of data when data was recorded.<br>What was said to happed and what actually happened may differ.
[69] Ding et al. (2021) Do-not-resuscitate decision making for terminally ill older patients in the emergency department: An explorative, descriptive inquiry	To explore factors influencing DNR decision-making among family members of terminally ill older	Qualitative, descriptive study.<br>12 family members.<br>Emergency department of a tertiary hospital in China.<br>Interviews between	Not within the focus of the study.	Not within the focus of the study.	Family members made DNR decisions based on factors including subjective perception of family members,	Not within the focus of the study.	Not within the focus of the study.	Greater focus on the willingness of patient family members to make DNR decisions where the patient is older, has more severe diseases, or has comorbid conditions.	Purposive sampling used may result in bias.<br>Single emergency department.<br>Only one female patient

of Chinese family members.<br><br>China.	adults in the emergency department.	October 2019 and June 2020. Conventional content analysis. Ethical approval.			conditions of the terminally ill older adults, external environmental factors and internal family factors.			Greater attention to the needs of DNR patients' and their family members to improve quality of life. Need to promote and raise understanding among family members to create effective communication for DNR discussions.	included in the study. Only represent family members.
[52] Dzeng et al. (2015) Influence of institutional culture and policies on do-not-resuscitate decision making at the end of life.<br><br>United States of America and United Kingdom.	To explore how physicians' approaches to DNR decision making at the end of life are shaped by institutional cultures and policies surrounding patient autonomy.	Exploratory qualitative study. 58 internal medicine physicians. Four academic medical centers (3 USA, 1 UK). Interviews from March 2013 to January 2014. Thematic analysis. Ethical approval.	Not within the focus of the study.	Discussion with patient or surrogate from a goal of care perspective and recommend against resuscitation or focus on comfort care without explicitly mentioning resuscitation. Those who prioritised autonomy found ways to balance autonomy and the law	A hospital's culture and policies' prioritisation of autonomy versus best interest appears to influence the way in which physician trainees conceptualise patient autonomy. The degree of choice influences recommendations physician trainees were willing to	Not within the focus of the study.	At each hospital, physicians' ethical attitudes toward DNR decision making reflected its hospital's policy position regarding its prioritisation of autonomy versus best interest.	Policies more oriented toward best-interest decision making might allow physicians the leeway to shift their focus from a discourse of choice to one of care and compassion.	Urban hospitals only. Interviews may not reflect actual practices as focus on opinions, attitudes, and perceptions of clinical practice.

				and making unilateral clinical decisions when they thought they were appropriate.	offer regarding DNR decision making.				
[51] Einstein et al. (2015) Dying for advice: Code status discussions between resident physicians and patients with advanced cancer-a national survey.<br><br>United States of America.	To identify barriers hindering informed decision making for code status discussions	Cross sectional study.<br>358 medical residents.<br>Resident's programs in the United States of America (19 of the 387 programs participated).<br>Online questionnaire time period not specified.<br>Statistical analysis.<br>Ethical approval.	Not within the focus of the study.	46.7% of residents were likely to discuss an estimate of prognosis and the value of CPR with the patient.<br>Only 30% of residents were likely to offer a recommendation on CPR.<br>69% of residents who were unwilling to offer a recommendation stated that deference to patient autonomy prevented them from providing guidance.	46.7% of respondents reported they were likely or very likely to share prognostic information.	Not within the focus of the study.	Not within the focus of the study.	End-of-life communication is of increasing importance and there is an unmet need in training and practice.<br>Attending physicians should discuss advance planning early in the disease course.<br>Physicians, residents, and attendings alike should be expected to provide guidance on the utility of medical interventions, including CPR.<br>If unsure of what to recommend, they should seek guidance from	Self-reporting bias.<br>Findings may not be generalised to residents outside the United States of America due to training and cultural differences.

				66.8% felt they should discuss code status themselves while 20.6% preferred to defer to an attending.				expert resources which may in turn encourage oncologists to discuss end-of-life planning early.	
[59] El-Jawahri et al. (2015) A randomized controlled trial of a CPR and intubation video decision support tool for hospitalized patients.<br><br>United States of America.	To examine the impact of a video decision tool for CPR and intubation on patients' choices, knowledge, medical orders, and discussions with providers.	Prospective randomised trial study. 150 patients. Inpatient internal medicine services at two teaching hospitals in the USA. Participants randomised, intervention arm were shown a 3-min digital video regarding CPR and intubation, control arm usual care, conducted between March 2011 and June 2013. Statistical analysis. Ethical approval.	Not within the focus of the study.	Documented discussions regarding CPR and intubation were similar between the intervention and control arms prior to study enrolment (47 %/39%) but were higher among intervention versus control participants at discharge (81 %/43%).	Not within the focus of the study.	The percent of participants with orders to withhold CPR (57%/19%) and intubation (64%/19%) were significantly higher in the intervention versus control arm.	In the intervention arm, 54 participants stated they did not want intubation after viewing the video, and only two of these participants received intubation in the 1-year follow-up period. In the control group, 32 participants stated they did not want intubation at baseline and seven of these participants received intubation in the follow-up period. There were no instances of	A video decision support tool can inform patients' preferences regarding CPR and intubation, increase physician–patient conversations on these topics in the inpatient setting, leading to more accurate documentation of patients' preferences and the delivery of medical care that is consistent with patients' wishes.	Study included predominantly white patients recruited. Hospitals have a palliative care presence and a culture accepting of innovative approaches to addressing goals of care. It is possible that participants' preferences changed over time on future hospitalisations.

							participants in either arm receiving CPR among those who stated that they did not want this treatment at baseline.		
[67] El Sayed et al. (2013) End-of-life care in Toronto neonatal intensive care units: challenges for physician trainees.<br><br>Canada.	To explore the challenges for trainees when end-of-life decisions are undertaken and reflect on how they might influence such decision-making.	Qualitative design not specified.<br>12 physician trainees.<br>Neonatal-perinatal fellowship training program in Canada.<br>Interviews January to December 2009.<br>Content analysis.<br>Ethical approval.	Not within the focus of the study.	Not within the focus of the study.	Decision driven by three factors deciding what is in the best interests of the infant, arriving at a consensus with parents and considering the therapeutic options available.<br>No one should feel they made the decision it is a shared decision.<br>Need to consider cultural expectations around end-of-life discussions.	Not within the focus of the study.	Resources such as ethics consultation can be used.	Inadequate training in communication skills essential for end-of-life decisions.<br>Need for ethical guidelines in caring for critically ill.<br>DNR can give the impression to families that the attempt at resuscitation is likely to succeed.	Limited to one university programme and small sample size.

[36] Fan and Hsieh. (2020) The experience of do-not-resuscitate orders and end-of-life care discussions among physicians.<br><br>Taiwan.	To explore the DNR and end-of-life are discussion experience among physicians in Taiwan.	Qualitative design not specified.<br>16 physicians.<br>Hospital in Taiwan.<br>Interviews March 2016 to June 2017.<br>Thematic analysis<br>Ethical approval.	Patients did not consider DNR and found it hard to think about when their physical condition is good. Difficult to accept DNR when close to death. Physicians in the oncology or internal medicine departments tended to raise the issue when physical condition had started to decline, which ensured that patients and family members had time to think about it.	Family members had multiple roles, information receivers, collaborative decision-makers, blockers and the persons caring for patients. Family members took responsibility for decision-making when physical condition and consciousness status prevent decision-making. Family dynamics influenced discussions and decisions and differences in opinions caused conflict between	The information pack included diagnosis, prognosis, disease trajectory, life expectancy, process of dying, symptoms, treatment plans, limitations of medical/hospice/palliative care/CPR/life-sustaining treatment, DNR - its consequences/effects, side effects of CPR, goals of care/care planning. The contents of information were based on patients' and family members' needs and physical conditions.	Not within the focus of the study.	Physicians in non-hospice wards followed the decision for DNR or continuing CPR. If patients and family members did not accept a DNR order, then they gave CPR till the patient's death based on the law.	Physicians need to develop communication skills and sensitivity.<br>There is a need for educational trainings on establishing trust relationships with patients and family members, solving the barriers, delivering information, and dealing with emotional reactions.<br>Physicians need training to conduct a family conference.	One medical center and shared the same policy of discussion.<br>Physician reporting perceived views of patients.

				family members.					
[34] Fan et al. (2018) Allow natural death versus do-not-resuscitate: titles, information contents, outcomes, and the considerations related to do-not-resuscitate decision.<br><br>Taiwan.	To explore how allow a natural death versus do not resuscitate influence the willingness to sign a DNR order, information needs and consideration of the DNR decisions; the benefits and barriers of the DNR discussion in the general population.	Cross-sectional study.<br>524 adults.<br>Family medicine clinic in Taiwan.<br>Questionnaire, time period not identified.<br>Statistical analysis.<br>Ethical approval.	30.34% would make the decision when the patient's disease had progressed to the terminal stage and 27.48% when the patient was healthy.	84.16% had heard of DNR, 24.43% had signed a DNR order.	54.39% felt patients and families should make the DNR decision together a shared responsibility. 35.50% felt patients should make the decision by themselves.<br>DNR discussion should focus on reducing suffering, supporting arrangement of medical care as wished and reducing family burden.	Not within the focus of the study.	Not within the focus of the study.	General population prefer the title allow natural death rather than DNR.<br>When have all relevant information people are more willing to sign a DNR order.<br>Discussion of the DNR and end-of-life issues involve various skills.	Convenience sampling used.<br>Participants aware of DNR and were willing to participate in the DNR discussion, may not apply to participants who are not familiar with DNR.
[17] Gibbs et al. (2016) Themes and variations: An exploratory international investigation into resuscitation decision-making.	To examine international variation in clinicians' perception of DNACPR decisions and	Cross sectional study.<br>78 physicians.<br>43 countries represented.<br>Questionnaire time period not specified.	Not within the focus of the study.	88% of respondents had conversations at least 'half of the time' in making decisions.	Culture and religion are factors that influence decision making.<br>Individual autonomy versus	87% worked in a hospital where there was a method for communicating decisions to	23 of the 43 countries sampled were aware of national guidance on DNACPR decision-making.	Societies in which the patient's family has great involvement in their care tend to give less value to the autonomy of the individual	Survey of physicians who had previously published material on end-of-life care, so may have specific

United Kingdom.	implementation and explores which factors influence such variation.	Statistical analysis. Ethical approval not stated.			paternalistic attitude.	their medical colleagues in written format (in the notes, electronically or by completing a pre-printed document).	94% thought that there should be national policy/guidance for making resuscitation decisions to standardise decision-making; provide support for clinicians minimise the potential for conflict with patient's families; and legitimise clinical CPR decisions.	patient, families often tend towards attempts to preserve life. Cultural attitudes towards death play a large role in determining how DNACPRs are perceived. Ambiguity about how national law views decisions to withdraw or withhold treatment is an obstacle.	interest or experience. For the majority of countries sampled, only one or two respondents were obtained.
[53] Goodarzi et al. (2022) Knowledge, attitude and decision-making of nurses in the resuscitation team towards terminating resuscitation and do-not-resuscitate order.<br><br>Iran.	To evaluate knowledge, attitude, and decision making about do-not-resuscitate and termination of resuscitation among nurses in the resuscitation team.	Cross sectional study. 128 nurses. Emergency wards in two Iran hospitals. Questionnaire, April-Sept 2020 Statistical analysis. Ethical approval.	Not within the focus of the study.	Not within the focus of the study.	Only 37.5% had DNR knowledge. DNR knowledge had significant relationship with age, educational level. Malignancy and age were the most prevalent factors which encouraged staff decide on DNR.	Medical orders respecting DNR are not documented in patients' records (71.9%). Physicians verbally order DNR but document complete CPR in patients' records (74.2%).	Not within the focus of the study.	Nurses have significantly lower knowledge about the ethical codes of CPR probably due to the fact that healthcare authorities consider physicians responsible for making DNR decisions. Nurses with experience (15 years) had more positive attitude	Only two hospitals. Limited causality.

					Malignancy and old age are factors which encouraged nurses to decide on DNR.			towards DNR order.	
[49] Hadley. (2020) Managing Do Not Attempt Cardiopulmonary Resuscitation conversations in the community.<br><br>United Kingdom.	To explore palliative care clinical nurse specialists' experiences of having DNACPR conversations in the community and language used.	Qualitative autoethnography study.<br>6 clinical nurse specialists.<br>Community setting.<br>Interviews, time period not specified.<br>Thematic analysis.<br>Ethical approval.	Not within the focus of the study.	Often must manage the aftermath of patients and relatives discovering DNACPR decisions had already been made.	Influenced by observation of other health professionals communicating DNACPR decisions with patients.<br>Conversation using plain and simple language to avoid confusion.<br>Confidence to have DNACPR conversations was impacted by knowing and having a rapport with the patient.<br>Factors affecting DNACPR conversations included patient acceptance,	Not within the focus of the study.	Not within the focus of the study.	DNACPR decisions that do not include consent from patients and relatives and operates on the multidisciplinary team agreement is likely to be unsuccessful and inappropriate.<br>Good practice is to include patients and families in discussions.<br>Delayed conversations result in conversations taking place with unfamiliar professionals.	Small-scale study with only one group of health professionals from a single organisation.

					rapport, finding a time when there were less interruptions, younger patients; managing lack of acceptance and understanding among patients and relatives, confidence and training, one's own emotions and culture.				
[25] Harrington et al. (2020) From paper to paperless: Do electronic systems ensure safe and effective communication and documentation of DNACPR decisions.<br><br>United Kingdom.	To review and improve practice in relation to DNACPR documentation and communication on gerontology wards in the trust.	Single-centred, prospective study.<br>259 patients, 26 healthcare professionals.<br>3 gerontology wards in a hospital.<br>Questionnaire, patient records and policy, Jan-June 2018.<br>Thematic analysis.<br>Ethical approval not specified.	66.8% of DNACPR decisions were made within 48 hours of the patient's admission.	Evidence of discussion with either the patient or relatives in 68.7% of cases.	Not within the focus of the study.	92.3% e had an explanatory e-DNACPR form.	Decisions needed to be made by a senior doctor, with a consultant countersignature.	Introducing electronic documentation for DNACPR is complex and safe and effective implementation requires construction of systems that enable high quality record keeping and timely communication.	Suitability of thematic analysis to questionnaire data.<br>One hospital and only three wards.

[22] Hendriks et al. (2022) Shared decision-making in advance care planning among hospitalized older COVID-19 patients: a multicenter, retrospective cohort study.<br><br>Netherlands.	To describe the practice of shared decision-making regarding code status during the COVID pandemic.	Retrospective cohort study. 275 COVID patients. 2 large hospitals. Electronic health records, Feb-April 2020. Statistical analysis. Ethical approval.	95% of the patients, it was documented that the patient was informed about code status in current hospital-admission.	Patient participation in the decision-making process was described in 48% of the cases.	Patients without treatment limitations, key elements for decision-making were more frequently missing	19% of patients had the decision-making process documented.	Not within the focus of the study.	Patients are not involved in a fully finalised decision-making process including careful documentation. Incorporating the key elements of shared decision-making in the mandatory code status form could improve attainment of a shared decision.	Focus on what was documented in the electronic health record which may not be the representation of the actual conversation that took place. Early phase of COVID.
[47] Holland et al. (2013) Barriers to involving older people in their resuscitation decisions: The primary-secondary care mismatch highlights the potential role of general practitioners.<br><br>United Kingdom.	To determine current clinical practice for patient involvement in resuscitation decision making in a sample of primary and secondary care clinicians and explore perceived barriers.	Mixed methods study. 24 primary and secondary care clinicians. Primary and secondary care in the UK. Questionnaire open and closed questions, time period not specified. Statistical analysis. Ethical approval.	Not within the focus of the study.	Hospital-based clinicians discuss resuscitation decisions with patients less than 25% of the time.	Patients' mental/cognitive function or lack of understanding was seen as the most important barrier to their involvement. Did not discuss resuscitation decisions if believed it would be distressing, or person lacked capacity. Setting for the	Not within the focus of the study.	Not within the focus of the study.	Resuscitation decisions are often made by clinicians in charge of patients' acute care, with views of close relatives considered.	Small sample size from one area of the UK. Non-validated questionnaire. Data saturation not reached, and number of responses not identified for open questions.

					discussions was a barrier as issues such as lack of privacy and the difficulty in finding appropriate time.				
[44] Hurst et al. (2013) Including patients in resuscitation decisions in Switzerland: From doing more to doing better.<br><br>Switzerland.	To explore physicians' justification for CPR/ DNAR orders and decisions regarding patient inclusion and how they initiated discussions with patients.	Cross sectional study.<br>61 residents in charge of 206 patients.<br>Six wards of University Hospitals.<br>Questionnaire, April 2004 - May 2005.<br>Statistical analysis.<br>Designated as quality control and exempted from full ethics committee review.	Not within the focus of the study.	52.8% DNAR had been discussed with the patient.	Diseases most frequently reported as determining a DNAR order were cancer, mostly described as untreatable, advanced, or terminal, heart disease, or the presence of multiple disorders.	21.2% contained a DNAR order.	Not within the focus of the study.	Residents used arguments based on respect for patient autonomy and decision-making capacity, as well as arguments based on their own clinical assessment of the situation, to justify including or not including patients in DNAR decisions.	Dated data 2004/2005. Recall bias is possible due to the delay between DNAR decisions and interviews. Decisions made at earlier stages in the patient's management tended not to be viewed as requiring revision.
[65] Ismail et al. (2015) Misunderstanding of the term "DNR" in a Middle Eastern teaching hospital.<br><br>Bahrain.	To measure the perception amongst the acute specialties of what constituted DNR orders.	Cross sectional study.<br>50 doctors.<br>University Hospital Bahrain.<br>Questionnaire, time period not specified.	Not within the focus of the study.	Not within the focus of the study.	46% physicians believed that the DNR decision lies with the doctor.<br>20% thought it is a family decision.	Not within the focus of the study.	98% of physicians thought that there should be a DNR policy and 96% thought there should be legislation regarding DNR.	Ambiguity in the interpretation of DNR, misinterpreted as being a decision on the management of the patient in the period before a cardiac arrest.	Non-validated questionnaire. Culturally respondents tend to give answers they believe will please the investigator.

		Statistical analysis. Ethical approval.			34% thought that the decision should be made by both the family and physician.			Involving the family in DNR decisions was found to be socially and culturally unacceptable from an Islamic perspective, doctors should make the decision as a team, and keep the family fully informed.	
[60] Khalaileh. (2014) Jordanian critical care nurses' attitudes toward and experiences of do not resuscitate orders.<br><br>Jordan.	To explore Jordanian critical care nurses' attitudes towards and experiences of DNR decisions in clinical practice.	Cross-sectional study. 111 nurses. Intensive care units in three government hospitals in Jordan. Questionnaire, time period not specified. Statistical analysis. Ethical approval.	Not within the focus of the study.	87% deemed that DNR decision-making processes should involve the physician and 67% the patient's family. 60% thought that nurses should also be involved in DNR decision-making processes.	Not within the focus of the study.	Regarding the documentati on of DNR orders in nursing notes, 81% preferred a coding system to a written statement.	74% emphasised the need for a clear DNR policy.	There is potential for the physician to be authoritarian or paternalistic and make decisions without respecting the patient's autonomy. A standard DNR form should be kept in the patient's medical record.	Convenience sampling used.
[37] Liu et al (2014) Taiwanese parents' experience of making a "do not resuscitate"	To explore the parental experience of making a "do not	Qualitative descriptive study. 16 parents.	Most of the children had been hospitalise d for a	All of the participants were approached to sign the	Factors influencing parents' decision to sign the DNR	Not within the focus of the study.	Not within the focus of the study.	Father mostly the one who led the discussion, and it was he who finally bore	Most children had a chronic disease, and the presence

decision for their child in pediatric intensive care unit.<br><br>Taiwan.	resuscitate" (DNR) decision for their child who is or was cared for in a pediatric intensive care unit.	Child was presently or had been a patient in the pediatric intensive care unit of a medical center in Taiwan. Interview, time period not specified. Thematic analysis.	period of time and the child's health had deteriorated prior to their parents being approached.	DNR form by a physician who was involved in their child's care.	form "can't bear any more", "poor future", and "health professional's knowledge, explanation and attitude". After signing parents feel a mixture of frustration, guilt, conflicting hope, regret and relief.			the responsibility to sign. Parents retain some hope for their child, even though they signed the DNR form. Doctors and nurses need to sensitively explain both the pros and cons of signing a DNR form, patiently wait for parents to assimilate the information and avoid rushing to a final decision and signature.	of chronic disease can impact parents' decision-making.
[38] Low et al. (2014) Palliative care staff's perceptions of do not attempt cardiopulmonary resuscitation discussions.<br><br>United Kingdom.	To explore clinician experiences of discussing DNACPR decisions with patients in a specialist palliative care inpatient and community setting.	Qualitative design not specified.<br>11 staff (6 nurses 5 medical).<br>Scottish hospice.<br>Interviews, time period not specified.<br>Thematic analysis.<br>Ethical approval.	Patient discharge from inpatient was a significant prompt for initiating a DNACPR discussion.<br>In the community change in a patient's condition was a significant trigger for	Not within the focus of the study.	Causing patients distress was the main inhibiting factor.<br>Patient characteristics inhibit conversations; younger, anxious, those who find it difficult to discuss their future and end-of-life	Not within the focus of the study.	Not within the focus of the study.	It most helpful to discuss DNACPR in the context of a wider end-of-life discussion.<br>Most patients deal with these discussions much more positively than staff anticipate.	Conducted with staff from one hospice only.<br>Short duration of interviews max 35 minutes.

			initiating a DNACPR discussion.		issues, those with an unrealistic view of their disease and those who have not come to terms with their prognosis. A strong patient relationship actually made it more difficult to discuss DNACPR, as an emotional connection could cloud judgment, or relating to a patient more strongly.				
[50] MacCormick et al. (2018) Resuscitation decisions at the end of life: Medical views and the juridification of practice.<br><br>United Kingdom.	To analyse the practice of resuscitation decision making on hospital wards from the perspectives of doctors.	Ethnographic study.<br>36 participants (9 patients, 11 relatives, 11 doctors, 5 staff members}.<br>Two acute medical wards in a district general hospital in England.<br>280 hours of observation	Not within the focus of this study.	Doctors acknowledged the need to involve patients and/or their families in resuscitation decisions, but occasions when this did not occur. Doctors hold varying	Doctors described feeling under pressure to have discussions about resuscitation with all patients soon after their admission. They acknowledge	Not within the focus of this study.	Not within the focus of this study.	There is a benefit in having a structure to support clinicians, patients and relatives in discussing and navigating decisions around care at the end-of-life in line with the	Study conducted in two wards in one hospital. Small sample of doctors. Focus was more on end-of-life care rather than resuscitation.

		observation and individual interviews in June 2014 end date not specified. Thematic analysis guided by a constructivist grounded theory approach. Ethical approval.		perspectives about what involvement requires.	d what a sensitive topic resuscitation could be and the difficulty in having such discussions with patients and relatives whom they had never met before.			patient's wishes and preferences.	
[56] Madadin et al. (2019) Clinicians' attitudes towards Do-Not-Resuscitate directives in a teaching hospital in Saudi Arabia.<br><br>Saudi Arabia.	To explore the influencing physicians' DNR decisions in King Fahd University Hospital.	Cross sectional study.<br>36 physicians. Medical and surgical ICUs in Saudi Arabian university hospital. Questionnaire time period not specified. Statistical analysis. Ethical approval.	Not within the focus of this study.	The National Policy's procedures include communicating the decision to the family or patients and clarifying that it is purely a medical decision.	Religious beliefs play a role in their decision-making. Clinical data important such as comorbidities, age, and previous ICU admissions.	Not within the focus of this study.	Not within the focus of this study.	Physicians noted that cultural standards and religious beliefs play a role in their decision-making but had less of an effect as compared to other clinical data such as comorbidities, age, and previous ICU admissions.	One hospital.
[73] McLawhorn et al. (2016) Integrating a question prompt list on an inpatient oncology unit to increase prognostic awareness.	To increase prognostic awareness by promoting patient–provider dialogue	Quantitative, Plan-Do-Study-Act cycle study.<br>152 admissions pre-implementation (128 patients)	Not within the focus of this study.	An increase was seen in the percentage of patients with active DNR orders and in hospice	The increase in the number of DNR orders and hospice referrals after inclusion of the	Not within the focus of this study.	Not within the focus of this study.	A prognosis-focused discussion aid brochure is an inexpensive and simple way to improve patient–provider	One hospital. No research methodology identified. Only descriptive statistics.

United States of America.	through use of a communication aid at the end of life.	196 admissions post-implementation (166 patients). Two inpatient oncology units in a tertiary medical center USA. Medical records reviewed for 3 months pre/post implementation Nov 2014 - Jan 2015 (pre) April - July 2015 (post). Statistical analysis. Ethical approval not specified, classed as quality improvement.		referrals after a discussion aid was included in admission packets.	discussion aid in admission packets supports improved patient–provider discussion and patient improved prognostic awareness.			dialogue, with potential to begin improving prognostic awareness.	
[41] Miller and Dorman. (2014) Resuscitation decisions for patients dying in the community: A qualitative interview study of general practitioner perspectives.	To investigate how general practitioners think and feel about making and communicating Do Not Attempt Cardiopulmonary	Qualitative design not specified. 10 general practitioners. Urban and rural area of Southern England. Interviews Sept to Dec 2011.	The general practitioners felt that the patient must understand that they are, or will be, dying, before it is possible to	General practitioners feel they should discuss the decision with the patient but have anxieties about this. General practitioners	General practitioners feel they should discuss the decision with patients who have capacity but are fearful of removing hope.	Not within the focus of this study.	Not within the focus of this study.	General practitioners would benefit from clearer guidance on when an attempt at resuscitation is unlikely to be successful, especially in non-malignant disease.	Small sample size.

United Kingdom.	Resuscitation decisions for patients dying in the community.	Interpretative phenomenological analysis. Ethical approval.	discuss a DNACPR decision.	vary widely in how much they guide patients and families in decision-making.				Team discussions including Gold Standards Framework meetings can give confidence and support making difficult end-of-life decisions.	
[58] Moaed et al. (2019) Factors influencing Do-Not-Resuscitate status in children during last month of life: Single institution experience.<br><br>Israel.	To evaluate factors that may influence the process of decision-making regarding Do-Not-Resuscitate status.	Retrospective study.<br>79 patients (109 hospitalisations).<br>Pediatric Oncology/Hematology unit in a childrens hospital in Israel.<br>Medical files of all patients who died Sept 2011 to Aug 2017.<br>Statistical analysis.<br>Ethical approval not specified.	Not within the focus of this study.	Not within the focus of this study.	In three documented cases on DNR status, the parents refused to give positive consent.	Written mention of DNR orders in 56 of 79 (70.9%) cases.	Not within the focus of this study.	Clarification of DNR status allows timely planning of the best treatment for actively dying children, facilitating the avoidance of futile and unnecessary medical interventions/investigations.<br>Clarified DNR status helps to lessen the psychologic stress imposed on medical and other staff treating the dying child.	One institution only.<br>Small sample size.<br>Limited to information documented in the medical records.
[62] Myers and Matthias. (2020) Nursing facility residents' cardiopulmonary	To understand the perspectives of	Qualitative design not specified.<br>30 participants (14 healthcare	Not within the focus of this study.	If an initial attempt to educate a resident is rejected or	Unrecognised and unaddressed differences in perception	Not within the focus of this study.	Not within the focus of this study.	Advance care planning communication models and training should	Participants selection process, residents with a

resuscitation decisions.<br><br>United States of America.	healthcare providers and nursing facility residents regarding CPR decisions.	professionals and 16 patients).<br>Five urban nursing facilities in USA.<br>Constant comparative analysis.<br>Interviews March-Dec 2018.<br>Ethical approval.		does not result in a decision change from CPR to DNR subsequently more information is offered at periodic intervals, with the hope that the resident will become receptive at some future point in time.	between residents/providers can impact CPR decisions.<br>Before attempting to elicit a resident's goals, values, preferences, healthcare providers must understand the resident's viewpoint on health status and prognosis.			be designed not only to explore nursing facility residents' goals, values, and preferences, but also to elicit any underlying differences in perceptions that may affect communication.	history of indifference or resistance to engaging in advance care planning communication with nursing facility staff may have been omitted.
[27] O'Brien et al. (2018) Do-not-attempt-resuscitation (DNAR) orders: Understanding and interpretation of their use in the hospitalised patient in Ireland. A brief report.<br><br>Ireland.	To examine current understanding of the term, do-not-attempt-resuscitate decision-making surrounding DNAR and awareness of current guidelines.	Mixed methods study.<br>519 healthcare workers.<br>Urban hospital Ireland.<br>Questionnaire open and closed questions.<br>July-Dec 2014.<br>Statistical analysis.<br>Ethical approval.	Preference for discussion of DNAR following admission of the patient to hospital secondary to their life-limiting illness among all groups.	Not within the focus of this study.	Not within the focus of this study.	Not within the focus of this study.	Not within the focus of this study.	Continued misunderstanding and overinterpretation of DNAR orders. Further collaboration and information are required for meaningful advance care plans.	One hospital.<br>Questionnaire not validated.<br>No evidence of qualitative data analysis or findings.
[40] Olver and Eliott. (2016) Translating into	To ascertain the opinion of patients	Qualitative design not specified.	The ideal timing of a DNR	Patients saw DNR decisions as	Patients favoured having time	The form for recording	Not within the focus of this study.	Documentation of the DNR decision should	45.1% of the participants were not

practice cancer patients' views on do-not-resuscitate decision-making.<br><br>Australia.	with cancer about their preferences for how and when DNR discussions occur.	51 patients. Urban hospital Australia. Interviews time period not specified. Thematic and discourse analysis. Ethical approval.	discussion would differ between patients. Standardising the timing and format of DNR discussion to fit hospital culture where resuscitation is the default option may distress patients who are not ready for such a discussion, and/or subject them to inappropriate interventions.	their decision as an autonomous adult, but needed medical facts about their condition, and the likelihood of the success of resuscitation, to make a rational decision.	to discuss their decision with medical staff and family and recognised that this decision could change over time and altered circumstances.	the instructions on DNR should be flexible enough to accommodate a spectrum of possibilities.		be flexible, allowing a narrative written by a patient's doctor and summarising a patient's current understanding of treatment choices and prognosis.<br>If a patient is not ready to make a DNR decision, the doctor should leave a medical directive based on a clinical judgment about the futility of resuscitation, clearly providing instruction for nursing staff.	considered near the end of their lives.
[46] Pettersson et al. (2018) Perspectives on the DNR decision process: A survey of nurses and physicians in	To investigate how important and how likely nurses and	Descriptive correlational study. 216 participants (132 nurses and 84 physicians).	Not within the focus of this study.	Almost half reported it not likely that the patient would be involved in	57% reported providing information to the patient was important.	The vast majority thought it both important, and likely to happen,	Not within the focus of this study.	Nurses and physicians need to talk openly about their different perspectives on DNR decisions,	Not all respondents answered every question.

hematology and oncology.<br><br>Sweden.	physicians considered various aspects of the DNR decision process, regarding participation, information and documentation.	Hematology and oncology units across 7 hospitals in Sweden.<br>Online questionnaire Feb-Oct 2017.<br>Statistical analysis.<br>Ethical approval.		the decision on DNR.<br>21% found it unimportant to inform patients of the DNR decision.	21% stated that this was likely to happen.<br>There were differences between nurses and physicians, regarding participation by and information to patients and relatives.	that the care team would be informed of the decision, and that the decision would be clearly documented.		so they can develop a deeper understanding of the decisions, especially in cases where they disagree.<br>Patients and relatives benefit from receiving the same information from all caregivers.	Non validated questionnaire.
[57] Ramages and Cheung. (2018) Why do older people refuse resuscitation? A qualitative study examining retirement village residents' resuscitation decisions.<br><br>New Zealand.	To investigate the resuscitation preferences of older New Zealanders in a retirement village or residential care setting and reasons for these preferences.	Qualitative design not specified.<br>37 participants.<br>Two retirement villages in New Zealand.<br>Interviews time period not specified.<br>Thematic analysis.<br>Ethical approval.	Not the focus of this study.	Not the focus of this study.	Factors that influenced decision were the wish for a natural death; advanced age; and a realistic awareness about the consequences of resuscitation.	Not the focus of this study.	Not the focus of this study.	People can make reasoned decisions about resuscitation based on balancing their subjective estimations of quality of life and the presumed consequences of resuscitation.<br>It is important to educate people on the potential outcomes of resuscitation and explore and document their reasoning when discussing resuscitation preferences so	Small sample size.<br>No baseline data on current health status, quality of life, religious or spiritual beliefs, which may have had an influence on their resuscitation decisions.

								their wishes can be respected.	
[42] Saltbaek et al. (2020) Cancer patients, physicians, and nurses differ in their attitudes toward the decisional role in do-not-resuscitate decision-making.<br><br>Denmark.	To explore cancer patients' and health care professional s' attitudes regarding DNR decision-making authority and timing of the decision.	Cross sectional study.<br>904 patients, 59 physicians and 160 nurses.<br>University hospital in Denmark.<br>Questionnaire November 2011.<br>Statistical analysis.<br>Ethical approval.	Attitudes of patients and physicians differed regarding both the decision-making authority and timing of the DNR decision.	Almost half of cancer patients and most of the physicians and nurses favoured collaborative decision-making.<br>In cases of disagreement both patients and physicians felt it was their right to make the ultimate decision.	For most documented DNR conversations it was unclear who had initiated the discussion and how the decisional power was shared.	A conversatio n about the DNR order between the patient and physician was documented in 60% of cases.	Not within the focus of this study.	There is a need for a greater awareness and discussion on decisional role and timing among both health care professionals and the public.	One hospital. Physicians choose who to invite.
[45] Schluep et al. (2020) A cross-sectional investigation of communication in Do-Not-Resuscitate orders in Dutch hospitals.<br><br>Netherlands.	To determine the prevalence of Do Not Resuscitate orders and describe recollection of CPR-directive conversation s.	Nationwide multicentre cross-sectional observational study.<br>1136 patients (13 hospitals).<br>Hospitalized patients at risk for suffering in-hospital cardiac arrest.<br>Questionnaire Jan-Feb 2019.<br>Statistical analysis.	Not within the focus of this study.	55.8% Patients recalled speaking to a health care professional about their CPR-directive.	Patients should be more involved in CPR counselling and physicians should focus on correct patient understandin g of directive which should be documented.	CPR directive was documented in 91.2% of medical records.	Not within the focus of this study.	CPR directive counselling should focus more on patient involvement and their understanding.	Data only on day per hospital. Non validated questionnaire .

		Ethical approval.							
[74] Sritharan et al. (2017) Doctors' attitudes regarding not for resuscitation orders.<br><br>Australia.	To explore doctors' attitudes regarding the discussion and writing of not for resuscitation orders and identify potential barriers to the completion of orders.	Cross sectional study.<br>107 doctors.<br>Urban tertiary hospital in Australia.<br>Questionnaire over 2 months in 2015 (unspecified).<br>Statistical analysis.<br>Ethical approval.	Not within the focus of this study.	Seventy-eight per cent of doctors had written an order.<br>Order best done by the treating doctor.<br>Participants thought the advance care planning team and the palliative care should conduct discussion.<br>Best to include patients, family and by general practitioners.	Predominant reason for order was medical reasons (terminal illness, futility, poor prognosis), patient/family wishes/request, goals-of-care discussions (quality of life, functional status, elderly, multiple comorbidities). Most doctors indicated they would require the consent of the patient, the power of attorney, a spouse, partner or other family and/or the nurse manager to	Not within the focus of this study.	Not within the focus of this study.	Most doctors (90%) believed the writing of orders would result in their patient receiving suboptimal care by other staff.<br>Very few orders currently written.	One hospital only.<br>Convenience sample.

					write an order.				
[54] Sterie et al. (2021) It's not magic: A qualitative analysis of geriatric physicians' explanations of cardio-pulmonary resuscitation in hospital admissions.<br><br>Switzerland.	To examine how CPR is explained in geriatric rehabilita-tion hospital admission interviews and circumstanc es in which physicians explain CPR and the content of these explanations .	Qualitative design not specified.<br>51 admissions.<br>One rehabilitation hospital in Switzerland.<br>Patient admission interviews June 2017 to January 2018.<br>Thematic content analysis.<br>Ethical approval.	Not within the focus of this study.	There is an expectation that CPR has been discussed. Present the topic as already known to the patient. While making an informed decision is the goal there is an underlying assumption that the decision has already been made and the task is simply to retrieve this decision and document it.	CPR was discussed in 43 of the 51 recorded admission interviews. Where the risks and outcomes of CPR were discussed, they were provoked by the patients misunderstan ding of a question or uncertainty.	Not within the focus of this study.	Not within the focus of this study.	The scarcity and simplicity of CPR explanations highlight a reluctance to have in-depth discussions and reflect the assumption that CPR does not need explaining. Providing patients with accurate information about the outcomes and risks of CPR is incremental for reaching informed decisions and patient- centred care.	Only one rehabilitation hospital.
[23] Sterie et al. (2022) Do you want us to try to resuscitate: Conversational practices generating patient decisions regarding	To explore how physicians elicit patients' preferences about cardio-pulmonary	Qualitative design not specified.<br>41 admissions.<br>One acute geriatric facility in Switzerland.	Discussion of CPR occurred during the admission interview. It was frequently mentioned	Not within focus of this study.	Most commonly (18 cases) patients expressing a preference consisted of the physician referring to	Not within focus of this study.	Not within focus of this study.	Decisions about the relevancy of life-sustaining interventions need to take place in an adequate setting to allow for	Only one geriatric facility.

cardiopulmonary resuscitation.<br><br>Switzerland.	resuscitation during hospital admission interviews.	Patient admission interviews April 2017 to January 2018. Conversation analysis' Ethical approval.	in the beginning or at the very end.		an option and seeking patient acknowledge ment or confirmation. Patient preference requires the patient to specify the relevant course of action to give patient full decisional autonomy.<br>In 12 conversations patient pre-empted discussion of DNACPR.			patient participation. There is a need for communication training regarding involving patients in conversations about goals of care.	
[71] Taubert et al. (2018) Talk CPR: A technology project to improve communication in do not attempt cardiopulmonary resuscitation decisions in palliative illness<br><br>United Kingdom.	To evaluate whether video resources to convey the salient facts involved in CPR and DNACPR decisions for people with palliative and life-limiting illness were	Mixed method study.<br>100 healthcare professionals (25 each - nurses, junior doctors, consultants, general practitioners) and a patient/carer group (n=24). Service review in Wales.	Not within the focus of this study.	Frequency of discussing DNACPR in the previous 12 months: Nurses 15 (0-3 times), 5 (3-6), 3 (6-10), 2 (10+ times);<br>Junior doctors 9 (0-3 times), 3 (3-6), 4 (6-10), 9 (10+ times);	Not within the focus of this study.	Not within the focus of this study.	Not within the focus of this study.	there is a need for advance care planning shared decision tools. Videos, apps and websites are one way of facilitating understanding and technology may replace the traditional method of handing out numerous paper patient	One facility. Variability in access to internet and Wi-Fi in different parts of Wales.

	acceptable or not.	Five phases; patient/carer engagement event (n=24), baseline exercise and aspirations, design, video production and website design, evaluation (survey n=100) and focus group (n=14 patient/carer group).		Consultants 8 (0-3 times), 2 (3-6), 1 (6-10), 14 (10+ times); GPs 7 (0-3 times), 10 (3-6), 4 (6-10), 4 (10+ times).				information leaflets. Discussions about CPR/DNACPR need to be more routine and meaningful. Patients need to be educated about the need for such discussions.	
[61] Tseng et al. (2017) Factors associated with the do-not-resuscitate decision among surrogates of elderly residents at a nursing home in Taiwan.<br><br>Taiwan.	To determine the factors associated with the DNR decisions of elderly residents in Taiwan.	Cross sectional study. 213 family surrogates. Hospital affiliated nursing home in Taiwan. Questionnaire January 2011 and January 2012. Statistical analysis. Ethical approval.	Not within the focus of this study.	83% of DNR directives were made by family surrogates. 64.3% of family surrogates agreed that the whole family should participate in discussing DNR.	Factors associated with the DNR decision included having severe brain injury, pulmonary disease, cancer, Buddhism, and having discussed DNR with the resident. 51.2% of residents had discussed DNR among family members but	Only 11.3% of elderly residents had DNR signed by either residents or by family surrogates.	Not within the focus of this study.	For surrogates to make informed decisions about DNR medical professionals need to take into consideration factors that appear to be important in decision making such as religion, prior conversations about values regarding CPR/DNR, beliefs about death and illness severity.	Only one nursing home.

					82.6% not with resident.				
[31] Ur Rahman et al. (2013) Care of terminally ill patients: An opinion survey among critical care healthcare providers in the Middle East.<br><br>Middle East	To explore the effect of training background and seniority on do-not to resuscitate decisions in the Middle East.	Cross sectional study.<br>86 participants. Members of the Pan Arab Society of Critical Care.<br>Questionnaire October 2007 to January 2008.<br>Statistical analysis.<br>Ethical approval not specified.	The best time to discuss end of life issues was prior to patient getting severely ill was favoured in 60.5% of responses.	46.5% of respondent wanted physicians to have the ultimate authority in the DNR decision.	Religion plays a role in making DNR decisions 58.3%).<br>The importance of comfort during dying was priority for 45.3%, and ability to pray while dying was main concern for 52.3 %.	Not within the focus of this study.	Training background was a significant factor affecting the interpretation of the term no code DNR.<br>DNR was equivalent to comfort care (38.5%).	Training background and level of seniority did not impact opinion on most of end-of-life issues.<br>DNR is considered equivalent to comfort care among majority of Middle Eastern trained physicians.	Small sample size.
[39] Wagemans et al. (2017) Do-Not-Attempt-Resuscitation orders for people with intellectual disabilities: dilemmas and uncertainties for ID physicians and trainees: The importance of the deliberation process.<br><br>Netherlands.	To clarify the problems and pitfalls of non-emergency DNAR decision-making for people with ID, from the perspective of ID physicians.	Qualitative design not specified.<br>40 physicians/trainees and 12 experts.<br>Intellectual disability setting in the Netherlands.<br>Semi-structured individual interviews, focus group interviews and an expert meeting December 2009 to June 2012.	Suitable occasions for talking about DNAR were when the person moves into a residential setting, or at a care plan meeting, or when a severe decline occurs in health status.	The impact of the ideas and opinions of the relatives in the decision-making process was unclear.<br>Assessment of quality of life left to the relatives.	The most important considerations for physicians when issuing a DNAR order were longstanding chronic medical conditions like congenital physical defects and epilepsy.<br>Considerations like diminished	Physicians asked the relatives to sign the DNAR order, to confirm that they had seen or approved it.<br>Other felt it was the doctor's professional decision and they did not want to burden the relatives.	Most physicians thought choking was an accident and should be treated as a non-natural death, implying that the person should be resuscitated even if a DNAR order had been issued.<br>In some health care organisations, this was formally recorded in writing as an	Professionals should be trained in the uncertainties of decision-making and should have sufficient time to engage in discussions and come to a decision.<br>Advance care planning and clear organisational procedures would support this process.	Only ID physicians interviewed.<br>People with IDs and paid care staff were not involved in the study.<br>Only a few relatives participated in expert meeting.

		Grounded theory analysis. Ethical approval not specified.			life expectancy, advanced age and severe decline in health status were often mentioned.		exception in the DNAR procedure.		

Abbreviations -- CPR - CardioPulmonary Resuscitation; DNAR – Do Not Attempt Resuscitation; DNR - Do Not Resuscitation; DNACPR - Do Not Attempt CardioPulmonary Resuscitation; e-DNACPR – electronic Do Not Attempt CardioPulmonary Resuscitation; ID – Intellectual Disability; UK – United Kingdom; USA – United States of America.
